# Complications of Wounds of the Spinal Cord

**Published:** 1918

**Authors:** 


***Complications of Wounds of the Spinal Cord.*** By GUSTAVE ROUSSY. *La Presse Médicale*, March 2, 1918.

One of the chief facts learned from the study of spinal cord lesions is that the prognosis often depends more on the complications than on the medullary lesion itself. Very noteworthy improvements are possible in these cases where grave complications are avoided. The latter occupy the first place as a cause of mortality.

The author finds that meningo-myelitis is rarely found, that it is possible to avoid the occurrence of bed-sores, which he does not considerer to be a trophic disturbance of essentially medullary origin. He finds that pleuro-pulmonary complications are very frequent and constitute one of the principal causes of death in wounds of the spinal cord.

It was formerly thought that in these cases death from purulent meningitis or meningo-myelitis was a frequent cause of death but the author's experience has been quite the opposite. He finds at autopsy meningitic lesions perfectly delineated near the point of the wound—that a reaction of defense has been set up in the form of fibrous adhesions, which oppose the extension for any great extent from the inflammatory lesion. In other words, in the immense majority of cases there are not the local infectious complications which aggravate the prognosis of war wounds of the spinal cord, but rather the infective visceral complications such as the pulmonary complications.

He deprecates and deplores the death soon after operation, in cases with large wounds of the spinal cord where the operation took place the first few days after the injury. He thinks that these patients are still in a state of shock and resists with the greatest difficulty general anaesthesia, or even surgical intervention under local anaesthesia. As long as there is a good tolerance for foreign bodies in the intramedullary region of the cord, and a fibrous defense reaction is forming, he is in favor of waiting for the shock to pass of.

Bedsores are without doubt one of the gravest dangers which menace the patients with a spinal cord wound, but they can be avoided in most cases or at least arrested in their extension if they are treated as they occur.

The factors most potent in the causation of bedsores are prolonged compression with the attendant difficulty in circulation at the seat of the compressed joints— both due to the fact that it is impossible for the patient to change his position without assistance. Roussy does not consider the decubitus as a trophic disturbance of central medullary origin; but as a trophic disturbance of local origin, a result of mechanical disturbance, and of chemical irritation of the skin due to chemical changes caused by the dejections, and favored by the diminution of resistance of the skin because of the medullary lesion and the general state.

A patient with a wound of the spinal cord who has no loss of sphincter control should not have bedsores. A patient who has incontinence of urine will suffer fatally from this complication if proper, rigorous hygienic measures are not taken from the moment of injury.

In lesions of the chorda equina there are practically never any bedsores when there are no sphincter disturbances.

From the point of view of prophylaxis he takes steps to prevent bedsores by the following method :

It is indispensable to change the position of the patient hourly during the day and several times during the night. The physician or his assistants should themselves rigorously examine the patients daily to see whether or not their instructions have been punctiliously carried out. Air mattresses are indispensable for these patients as rubber rings give bad results causing bedsores over the trochanters from lateral pressure. Suspension beds are also defective.

The skin should be given an alcohol lavage daily, followed by complete drying and then powdered with talc or Lucas Championnière's powder.

Absolutely avoid soiling the skin by faeces or urine which are the principal sources of local infection. It is sometimes difficult to protect the skin from contact of faeces because there often exists a diarrhoea. Roussy, therefore, suggests in these cases that before transporting them, opiates should be given to cause constipation.

The factors favoring pleuro-pulmonary complications are many — those especially potent being, that the patients bear transportation very poorly even for a short time or for a short distance; and they are particularly sensitive to cold. In any case prolonged decubitus favors hypostatic pneumonia. When a patient lies for a long time on one side pulmonary lesions predominate on that side.

Frequent examinations of the chest are necessary, particularly when the patient presents any fever, either with or without cough.

